# Evaluation of Pulse Pressure as a Hemodynamic Marker of Cardiac Disease in Dogs and Horses Undergoing Pre-Anesthetic Assessment

**DOI:** 10.3390/ani16040569

**Published:** 2026-02-12

**Authors:** Ismar Lutvikadic, Dajna Preldzic, Dario Floriano, Klaus Hopster

**Affiliations:** 1 Department of Clinical Studies, New Bolton Center, School of Veterinary Medicine, University of Pennsylvania, 382 W Street Rd., Kennett Square, PA 19348, USA; khopster@vet.upenn.edu; 2 Department of Clinical Science, Veterinary Faculty, University of Sarajevo, Zmaja od Bosne 90, 71000 Sarajevo, Bosnia and Herzegovina; dajna.preldzic@vfs.unsa.ba; 3 Department of Clinical Studies and Advanced Medicine, Matthew J. Ryan Hospital, School of Veterinary Medicine, University of Pennsylvania, 2900 Spruce St., Philadelphia, PA 19104, USA; darioflo@vet.upenn.edu

**Keywords:** cardiac disease, dogs, horses, preanesthetic evaluation, pulse pressure

## Abstract

Pulse pressure (PP) reflects ventricular stroke volume and arterial compliance, but its utility as a marker of cardiac disease in animals is not well established. This study evaluated the association between PP and echocardiographically confirmed cardiac abnormalities in dogs and horses and assessed its potential in pre-anesthetic evaluation. Clinical and echocardiography examination of 20 dogs and 20 horses was sufficient for inter-group comparisons and assignments to a Cardiac group (echocardiographically confirmed cardiac disease) or a Control group (healthy animals). Non-invasive oscillometric blood pressure was measured, and PP was calculated. Animals with cardiac abnormalities showed significantly higher PP but not mean arterial blood pressure (MAP) compared with healthy subjects. In horses, PP was approximately 22 mmHg higher in the Cardiac group (*p* = 0.042), while in dogs, the increase was 25 mmHg (*p* < 0.001). Regression analysis confirmed cardiac status as an independent predictor of elevated PP (*p* = 0.001) with excellent and good discriminatory performance between healthy and diseased dogs (AUC = 0.90; 95% CI: 0.77–1.00) and healthy and diseased horses (AUC = 0.81; 95% CI: 0.61–1.00), respectively. These results suggest that PP may serve as a sensitive and practical hemodynamic indicator of underlying cardiac disease.

## Simple Summary

Careful cardiovascular assessment before anesthesia is important for reducing anesthetic risk in veterinary patients. Pulse pressure, defined as the difference between systolic and diastolic blood pressure, reflects how much blood the heart ejects with each beat and how elastic the arteries are. Despite its physiological relevance, pulse pressure is rarely used as a screening parameter in routine veterinary practice. In this study, pulse pressure was evaluated as a potential indicator of cardiac disease in dogs and horses undergoing pre-anesthetic assessment. Animals with echocardiographically confirmed cardiac abnormalities showed consistently higher pulse pressure values compared with clinically healthy animals, while mean arterial pressure remained similar between groups. These findings suggest that changes in pulse pressure may occur even when overall blood pressure appears normal. Because pulse pressure can be obtained easily from standard non-invasive blood pressure measurements in conscious animals, it represents a practical and low-cost addition to routine pre-anesthetic evaluation. Identifying unexpectedly increased pulse pressure in apparently healthy animals may prompt further cardiac investigation or encourage more cautious anesthetic planning. Overall, this study supports the use of pulse pressure as a simple, non-invasive tool to help detect occult cardiac disease and improve anesthetic safety in veterinary patients.

## 1. Introduction

Pre-anesthetic animal risk assessment is a cornerstone of veterinary anesthesia, designed to minimize perioperative complications and improve patient outcomes. A comprehensive evaluation typically includes assessment of body condition, hydration status, temperature, respiratory function, and cardiovascular status through pulse palpation and cardiac auscultation [1,2]. In both human and veterinary medicine, minimizing unnecessary diagnostics in low-risk cases will reduce costs, patient stress, and surgery delays. Laboratory testing and additional tests are therefore recommended primarily for geriatric patients or when disease is suspected [2,3]. Because anesthetic agents exert varying degrees of cardiovascular depression, identifying patients with preexisting cardiac abnormalities is essential [1,3]. Peripheral pulse palpation in conscious animals provides a rapid and low-cost means of assessing circulatory function, offering a crude estimation of blood pressure [2,4]. Nevertheless, noninvasive blood pressure measurement via Doppler or oscillometry is recommended to provide quantitative values and monitor trends beyond the qualitative limitations of palpation [2,5].

Under physiologic conditions, the difference between systolic and diastolic pressure (pulse pressure [PP]) reflects the volume of the ejected blood from the ventricle, but it may vary during disease [6,7]. Although its diagnostic value has not been fully explored, several studies suggest that PP monitoring in veterinary medicine may serve as a heart disease predictor, especially valve regurgitation [6,8]. In such conditions, diastolic arterial pressure (DAP) may decrease, and systolic arterial pressure (SAP) may increase due to activation of cardiac compensatory mechanisms [9,10,11]. In human patients, altered PP has been linked to a variety of cardiovascular disorders [12,13,14,15]. A widened PP (>60 mmHg) has been associated with aortic regurgitation, arterial stiffness, and hyperthyroidism, whereas a narrowed PP (<20 mmHg) may indicate heart failure, hypovolemia, or aortic stenosis [6,16]. Notably, each 10 mmHg increase in PP has been correlated with a 20% rise in cardiovascular risk in humans [15]. Additionally, elevated PP has been associated with tissue injury in vital organs, particularly the brain and kidneys, in experimental and clinical studies [12,17,18,19]. In contrast, in veterinary medicine, most previous research has focused on pulse pressure variation (PPV), a dynamic (beat-to-beat) measure that reflects changes in intrathoracic pressure and is primarily used to assess fluid responsiveness during mechanical ventilation [20,21,22]. While PPV is valuable intraoperatively for guiding fluid therapy, its use is limited to anesthetized, mechanically ventilated animals and does not provide information about baseline cardiovascular function [20,21,23,24,25]. In contrast, static PP, measured in conscious animals before anesthesia, could represent a simple, non-invasive screening parameter for underlying cardiac dysfunction, but its potential has received little systematic evaluation [6,8].

The objective of this study was to evaluate the relationship between PP and echocardiographically confirmed cardiac abnormalities in dogs and horses, and to determine whether PP can serve as a sensitive pre-anesthetic marker of cardiac disease. We hypothesized that animals with cardiac abnormalities would exhibit significantly higher PP compared with controls, whereas MAP would remain unchanged.

## 2. Materials and Methods

This study was approved by the Institutional Ethics Committee of the University of Sarajevo—Veterinary Faculty (approval number: 07-03-1044-2/25), and written informed owner consent was obtained for all animals.

### 2.1. Animals

A total of 20 dogs and 20 horses of various breeds, sexes, and ages were enrolled from clinical cases scheduled for sedation at the University of Sarajevo—Veterinary Faculty. None of the animals presented with acute illness, trauma, or pregnancy. Indications for sedation included mostly diagnostic procedures.

### 2.2. Protocol

All animals underwent a detailed physical examination, which included body condition scoring (BCS), body temperature, heart rate (HR), peripheral pulse quality assessment, heart auscultation, non-invasive blood pressure measuring (NIBP), respiratory rate, lower and upper airway auscultation, mucous membranes, determination of capillary refill time, hydration status assessment, mentation evaluation, abdominal palpation and auscultation, and overall condition assessment. NIBP was determined using an oscillometric device (Veterinary HDO Blood pressure Monitor S + B MedVet, GmbH, Babenhausen, Germany). The cuff width corresponded to 40% of either the front leg or the tail base circumference. Measurements were obtained at the antebrachium in dogs positioned in sternal recumbency, or on the tail base in standing horses. All measurements were performed by the same person (an anesthesiologist). Three consecutive NIBP measurements were obtained for each animal, and values were averaged. Following physical examination, transthoracic echocardiography was performed by an investigator blind to the group allocation. Echocardiography was conducted using an Esaote ultrasound device (MyLab30Gold Vet, Firenze, Italy) equipped with a 6–12 MHz phased-array transducer (PA122) for dogs and a 1–4 MHz phased-array transducer (PA230) for horses. In both species, echocardiographic measurements and calculations were performed in two-dimensional (B-mode) and time-motion (M-mode) modes, as explained by Boon [26] and de Madron [27]. An additional manually calculated parameter in dogs was left ventricular internal diameter in diastole (LVIDdN), normalized for body weight [28]. Color Doppler (CFM) was used to detect any turbulent blood flow at the level of the atrioventricular or semilunar cardiac valves [26]. The blood pressure and echocardiography were performed in conscious, unsedated animals. Animals with detected heart murmur and echocardiographically confirmed cardiac abnormalities were assigned to the Cardiac group, while the Control group consisted of animals with no murmur and without echocardiographic changes.

### 2.3. Data Collection

Data of interest for our study consisted of signalments (animal species, breed, age, and sex), HR, SAP, DAP, MAP, PP, heart murmur grade, and irregularity detected on echocardiography.

### 2.4. Statistical Analysis

The required minimum sample size was *n* = 7 dogs and *n* = 8 horses per group, which was exceeded in this study (*n* = 10 per group), yielding post hoc power of 0.95 and 0.85 for dogs and horses, respectively.

An a priori power analysis was conducted using G*Power (version 3.1.9.7) for a two-sample *t*-test, assuming an α = 0.05, two-tailed test, and power (1 − β) = 0.8. Based on preliminary data, an effect size of d = 1.2 was anticipated for PP between groups. No outliers for pulse pressure or mean arterial pressure were excluded from the analysis. All data were analyzed using IBM SPSS Statistics (version 29.0, IBM Corp., Armonk, NY, USA) and confirmed in GraphPad Prism (version 10.2, GraphPad Software, San Diego, CA, USA) for visualization. Statistical significance was set at *p* < 0.05 for all analyses.

Continuous variables (HR, SAP, DAP, MAP, and PP) were assessed for normality using the Shapiro–Wilk test and by visual inspection of Q–Q plots and histograms. All variables were normally distributed and are presented as mean ± standard deviation (SD). For graphical presentation, median and range were used in selected figures to illustrate data spread and individual variability. Homogeneity of variances between groups was verified using Levene’s test. For each species (dogs and horses), independent samples *t*-tests were used to compare MAP and PP between the Control and Cardiac groups. To account for multiple testing, the Holm–Bonferroni method was applied to adjust *p*-values. Cohen’s d was calculated as an estimate of effect size, interpreted as small (0.2), medium (0.5), or large (≥0.8). Ninety-five percent confidence intervals (95% CI) were reported for mean differences and effect sizes. To assess whether cardiac status independently predicted changes in PP and MAP after accounting for potential confounders, multiple linear regression models were constructed for each dependent variable (PP and MAP). The independent variables included cardiac status, age, sex, and species. Separate models were initially fitted for dogs and horses, followed by a combined species model to evaluate interspecies consistency. Model diagnostics included: examination of residuals versus fitted plots to assess linearity and homoscedasticity, Normal Q–Q plots of standardized residuals to confirm normality, and the Durbin–Watson statistic to evaluate the independence of residuals.

The diagnostic performance of PP in discriminating animals with cardiac disease from controls was evaluated using ROC curve analysis. The area under the curve (AUC) with 95% CI was calculated as a global measure of discrimination, interpreted as follows: 0.90–1.00 = excellent, 0.80–0.89 = good, 0.70–0.79 = fair, <0.70 = poor. The optimal PP cutoff value was determined by maximizing Youden’s Index (J = Sensitivity + Specificity − 1). Sensitivity, specificity, positive predictive value (PPV), and negative predictive value (NPV) were calculated at this cutoff. ROC analysis was performed separately for dogs and horses.

## 3. Results

No animals were excluded after enrollment. The overall demographic data and differences between the Cardiac and Control groups for horses and dogs are presented in Table 1 and Table 2. Recorded values of hemodynamic parameters in the Cardiac group are shown in Table 3.

The predominant echocardiographic finding in horses was aortic regurgitation, while in dogs it was mitral valve regurgitation. Other detected abnormalities in horses were: tricuspid regurgitation, aortic dilation, hypertensive cardiomyopathy, pulmonary regurgitation, and mitral valve regurgitation. Other detected abnormalities in dogs were: myxomatous mitral valve disease, aortic regurgitation, pulmonary regurgitation, aortic stenosis, and pulmonary stenosis. None of the animals in the Control group had audible heart murmurs. In the Cardiac group, heart murmur ranged from 1 to 4 and 2 to 5 out of 6 in horses and dogs, respectively.

**Figure 1:**
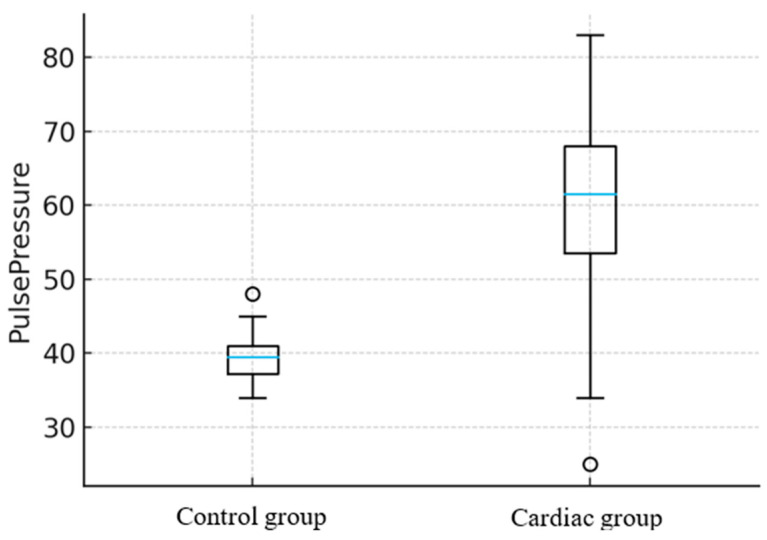
Comparison of pulse pressure (PP) values between healthy horses and those with detected cardiac changes. The median [blue horizontal line] (range [whiskers]) PP in the Control group was 40 (34–46) mmHg, while in the Cardiac group it was 62 (34–83) mmHg. PP was 22 mmHg higher in horses with cardiac changes compared to healthy animals (*p* = 0.042). Circle indicates outliers.

**Figure 2:**
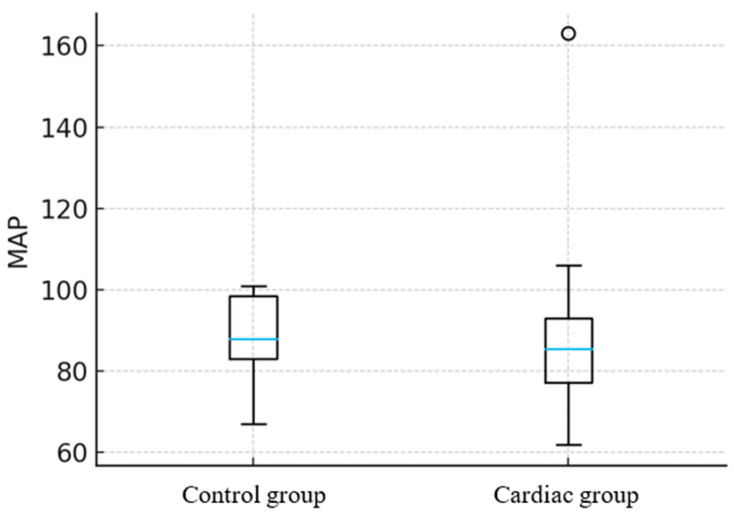
Comparison of mean arterial pressure (MAP) values between healthy horses and those with detected cardiac changes. The median [blue horizontal line] (range [whiskers]) MAP in the Control group was 89 (67–101) mmHg, while in the Cardiac group it was 87 (61–107) mmHg. MAP did not differ significantly between groups (*p* = 0.78). Circle indicates outliers.

Animals with documented cardiac abnormalities exhibited significant alterations in PP but not MAP. In horses, PP was approximately 22 mmHg higher in those with cardiac changes compared with clinically healthy animals (*p* = 0.042) (Figure 1), while MAP showed no statistically significant difference between Control and Cardiac groups, *p* = 0.78 (Figure 2).

**Figure 3:**
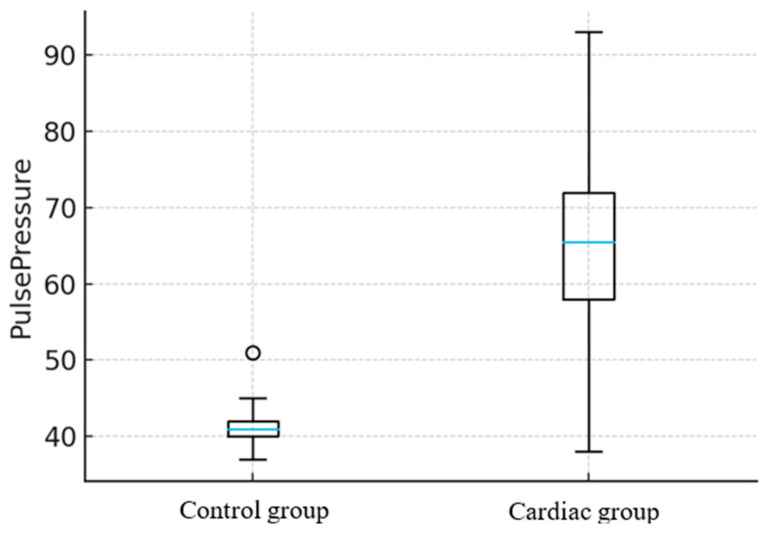
Comparison of pulse pressure (PP) values between healthy dogs and those with detected cardiac changes. The median [blue horizontal line] (range [whiskers]) PP in the Control group was 41 (37–45) mmHg, while in the Cardiac group it was 66 (38–93) mmHg. PP was markedly increased (25 mmHg) in dogs with cardiac changes compared to healthy animals (*p* < 0.001). Circle indicates outliers.

**Figure 4:**
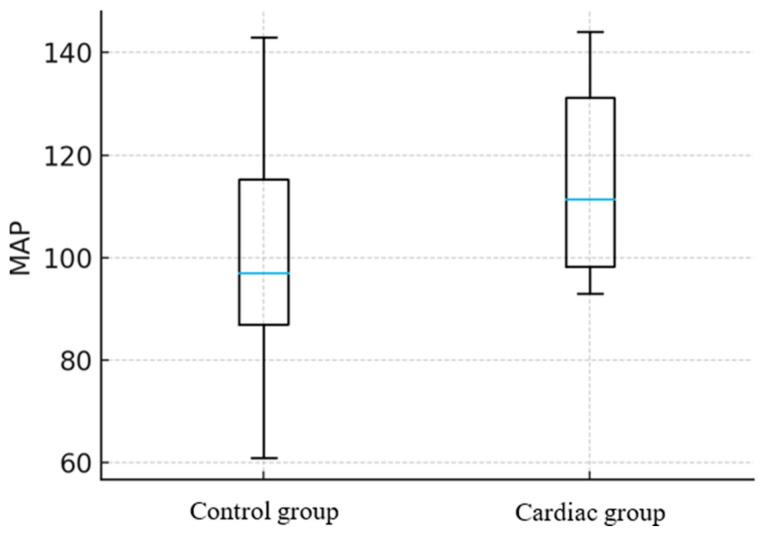
Comparison of mean arterial pressure (MAP) values between healthy dogs and those with detected cardiac changes. The median [blue horizontal line] (range [whiskers]) MAP in the Control group was 97 (60–142) mmHg, while in the Cardiac group it was 112 (93–143) mmHg. MAP did not differ significantly between groups (*p* = 0.59).

In dogs, PP was markedly increased (25 mmHg, *p* < 0.001) (Figure 3). Also in this group, the MAP showed no statistically significant difference between the Control and Cardiac groups, *p* = 0.59 (Figure 4). A combined linear model, which included both species, confirmed that the presence of cardiac changes was independently associated with elevated PP (*p* = 0.001). In contrast, species, age, and sex were not significant predictors. Overall, in the multiple linear regression model including cardiac status, species, age, and sex, the presence of a cardiac abnormality was independently associated with higher PP (β = 17.8 mmHg, SE 3.6, *p* < 0.001), whereas species, age, and sex were not significant predictors. In contrast, none of the predictors was significantly associated with MAP (all *p* > 0.2).

**Figure 5:**
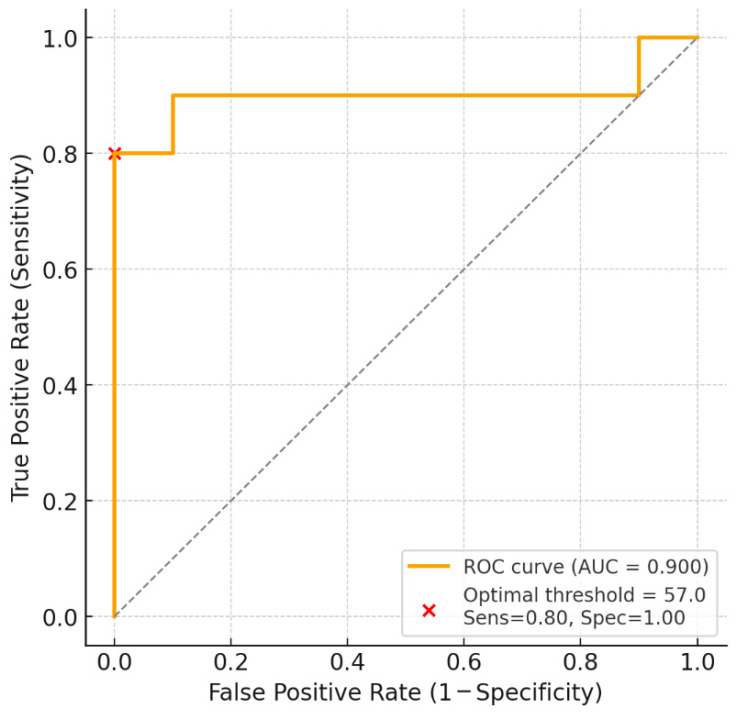
Receiver operating characteristic (ROC) curve illustrating the ability of pulse pressure (PP) to discriminate between healthy and diseased dogs. The curve shows the relationship between sensitivity (true positive rate) and 1 − specificity (false positive rate) across different PP thresholds. The area under the curve (AUC) is 0.90, indicating excellent diagnostic accuracy. The optimal threshold value for PP was 57.0 mmHg, providing a sensitivity of 0.80 and specificity of 1.00, as indicated by the red marker. The dashed diagonal line represents the line of no discrimination (AUC = 0.5).

**Figure 6:**
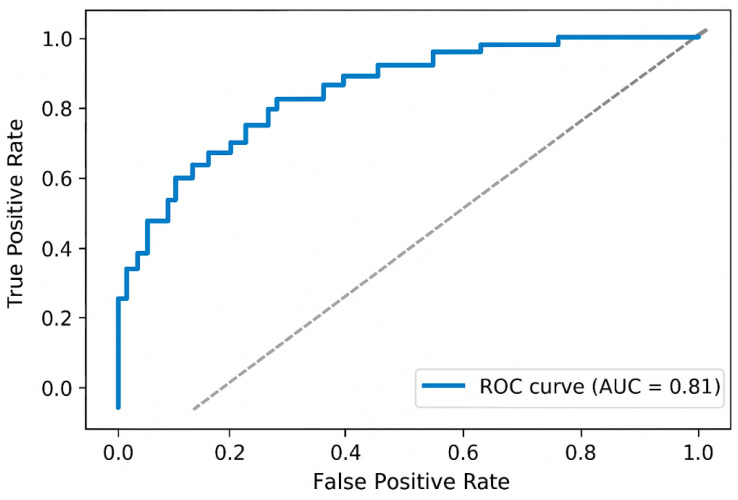
Receiver operating characteristic (ROC) curve illustrating the diagnostic performance of pulse pressure (PP) for detecting cardiac abnormalities in horses. The curve plots the true positive rate (sensitivity) against the false positive rate (1 − specificity) at various PP thresholds. The area under the curve (AUC) of 0.81 indicates good discriminative ability of PP to distinguish horses with and without echocardiographically confirmed cardiac abnormalities. The dashed diagonal line represents the line of no discrimination (AUC = 0.5).

Pulse pressure showed excellent discriminatory performance between healthy and diseased dogs (AUC = 0.90; 95% CI: 0.77–1.00) (Figure 5). The Youden-optimal cutoff was 57.0 mmHg, yielding a sensitivity of 80% and specificity of 100% in this dataset. ROC analysis using pulse pressure as a predictor yielded an AUC of 0.81 (95% CI: 0.61–1.00), suggesting good ability to differentiate between healthy and diseased horses (Figure 6).

## 4. Discussion

The present study demonstrated that PP was significantly higher in dogs and horses with echocardiographically confirmed cardiac abnormalities, while MAP remained unchanged. This finding supports its potential role as a sensitive indicator of chronic cardiac disease during pre-anesthetic evaluation. The effect was consistent across species, with a 22 mmHg increase in PP in horses and a 25 mmHg increase in dogs with cardiac disease, indicating a robust relationship independent of species, age, or sex. Based on power analysis, using 10 animals per group was more than sufficient to detect significant results, providing a safety margin and increasing power, allowing for extra subjects to cover possible exclusions. Our results align with observations from human medicine, where PP widening is strongly associated with aortic regurgitation, arterial stiffness, and ventricular dysfunction [13,14,15,29]. Similar to human patients, increased PP in animals with cardiac abnormalities likely reflects altered arterial compliance and increased stroke volume variation secondary to valvular regurgitation or structural cardiac changes [6,8]. In contrast, MAP remains relatively stable, likely due to compensatory adjustments in vascular resistance and heart rate [7,30]. Therefore, a stable MAP may mask underlying cardiovascular dysfunction, whereas elevated PP can serve as an early, more sensitive indicator of hemodynamic alteration.

In veterinary medicine, most previous work has focused on pulse pressure variation (PPV) as a dynamic indicator of fluid responsiveness during anesthesia [20,21,22], rather than as a static index of chronic cardiac disease. Animals with normal cardiac function maintain a normal PP (around 40 mmHg) through preserved ventricular function and arterial elasticity [6,31]. The present study expands this perspective by showing that baseline PP values in conscious animals may carry diagnostic significance. In terms of lesion specificity, aortic and mitral valve regurgitation appears particularly influential in widening PP, as backflow of blood into the ventricle and atria increases stroke volume and arterial pulse amplitude [8]. This can explain higher PP values in horses with aortic regurgitation and in dogs with mitral valve insufficiency, as both lesions lead to volume overload. In horses, similar findings were reported by Boegli et al. [8], who noted that non-invasive PP measurement could distinguish equine patients with aortic regurgitation from healthy controls. Our findings in dogs complement this evidence, suggesting that PP assessment might be incorporated as a practical screening tool for subclinical or compensated cardiac disease during routine pre-anesthetic evaluation. PP value in the cardiac group was slightly higher in dogs compared to horses. The smaller PP increase in horses is most consistent with greater arterial compliance, which limits pressure change for a given volume displacement according to the Windkessel model.

Clinically, these results reinforce the diagnostic value of integrating PP measurement into routine pre-anesthetic assessment, especially in geriatric or high-risk patients. Oscillometric blood pressure monitoring is widely available, rapid, and minimally invasive, making it suitable for routine use [5,7]. Detecting unexpectedly widened PP in an apparently healthy animal may warrant echocardiographic screening or modification of anesthetic protocols to mitigate perioperative risk [32]. Given that PP elevation may precede overt clinical signs, its use as a low-cost adjunct for cardiovascular screening could enhance anesthetic safety and early disease recognition.

Several limitations must be acknowledged. The Cardiac group included animals with heterogeneous lesion types, which may have exerted different effects on PP magnitude. All blood pressure measurements were obtained non-invasively, which, while clinically relevant, may introduce variability compared to direct arterial pressure monitoring. The measurement site (limb versus tail base) could influence the recorded PP values due to local differences in arterial compliance or vessel diameter. Inter-operator variability and subtle differences in cuff placement or tension may further affect accuracy. However, this potential effect was minimized by having the same investigator perform the physical examination in all animals. In addition, physiological factors such as age, excitement, restraint, or ambient temperature may have influenced vascular tone and, consequently, PP measurements. The highest PP values were observed in both young (dogs) and older (horses) individuals, while several geriatric horses exhibited comparatively low PP. These findings suggest that factors beyond chronological age influence PP in veterinary patients. Nevertheless, the incomplete characterization of vascular aging in animals remains a limitation when interpreting PP as a preanesthetic screening tool. Future studies using invasive pressure monitoring and larger, lesion-specific cohorts are needed to validate these findings and establish reference intervals for PP in different clinical contexts. We suggest a prospective multicenter validation and development of species-specific reference cutoffs.

## 5. Conclusions

Our results suggest that PP is a promising practical hemodynamic marker of underlying cardiac disease during pre-anesthetic assessment. Routine inclusion of PP measurement during pre-anesthetic cardiovascular assessment may facilitate early recognition of occult cardiac disease and enable more tailored anesthetic management. Further research with larger sample sizes, lesion-specific analysis, multicenter validation, and development of species-specific reference cutoffs is warranted to refine its diagnostic and prognostic applications.

## Figures and Tables

**Table 1:** The overall demographic data in two different animal populations included in the study (horses and dogs). The table is showing a variety of breeds, sexes, and ages. Ages are represented as mean ± SD.

Data	Horses (*n* = 20)	Dogs (*n* = 20)
Breed	Warmblood cross breeds (*n* = 11) Arabian horses (*n* = 5) Thoroughbreds (*n* = 3) Dutch warmblood (*n* = 1)	Mixed breeds (*n* = 5) Maltese dog (*n* = 3) German Shepherd Dog (*n* = 2) Pit bull (*n* = 2) Poodle (*n* = 1) Chihuahua (*n* = 1) Cocker spaniel (*n* = 1) Boxer (*n* = 1) Beagle (*n* = 1) Great Dane (*n* = 1) Bernese Mountain Dog (*n* = 1) Miniature pincher (*n* = 1)
Sex	Males (*n* = 15) Females (*n* = 5)	Males (*n* = 10) Females (*n* = 10)
Age	13.8 ± 5.2	7.7 ± 3.6

**Table 2:** The table presents a variety of breeds, sexes, and ages between the Cardiac group and the Control group of two different animal populations included in the study. Ages are represented as mean ± SD.

Data	Horses (*n* = 20)	Dogs (*n* = 20)
Breed		
Cardiac group (*n* = 10)	Warmblood cross (*n* = 8) Arabian horse (*n* = 1) Thoroughbred (*n* = 1)	Maltese dog (*n* = 2) Mixed breeds (*n* = 2) Beagle (*n* = 1) Poodle (*n* = 1) Cocker spaniel (*n* = 1) German Shepherd Dog (*n* = 1) Boxer (*n* = 1) Chihuahua (*n* = 1) Mixed breeds (*n* = 3) Pit Bull (*n* = 2) Bernese Mountain dog (*n* = 1) Miniature pinscher (*n* = 1) German Shepherd Dog (*n* = 1) Great Dane (*n* = 1) Maltese dog (*n* = 1)
Control group (*n* = 10)	Arabian horse (*n* = 4) Warmblood cross (*n* = 3) Thoroughbred (*n* = 2) Dutch warmblood (*n* = 1)	
Sex		
Cardiac group (*n* = 10)	Males (*n* = 9) vs. Females (*n* = 1)	Males (*n* = 7) vs. Females (*n* = 3)
Control group (*n* = 10)	Males (*n* = 6) vs. Females (*n* = 4)	Males (*n* = 3) vs. Females (*n* = 7)
Age		
Cardiac group (*n* = 10)	16.7 ± 3.2	8.8 ± 2.9
Control group (*n* = 10)	10.8 ± 5.1	6.5 ± 3.8

**Table 3:** Presentation of the mean (±SD) value of heart rate (HR), systolic arterial pressure (SAP), diastolic arterial pressure (DAP), mean arterial pressure (MAP), and pulse pressure (PP) in the Cardiac group.

Data	Horses (*n* = 10)	Dogs (*n* = 10)
Mean HR (bpm) (±SD)	58 ± 12	114 ± 14
Mean SAP (mmHg) (±SD)	130 ± 28	157 ± 27
Mean DAP (mmHg) (±SD)	72 ± 28	92 ± 26
Mean MAP (mmHg) (±SD)	91 ± 27	115 ± 22
Mean PP (mmHg) (±SD)	58 ± 17	65 ± 7

## Data Availability

Further specific data regarding each animal can be requested from the Authors.
